# Radiological Aspects in the Evaluation of Portland Cements with Fine Aggregate Additions

**DOI:** 10.3390/ma19163506

**Published:** 2026-08-19

**Authors:** José Antonio Suárez-Navarro, Miguel Angel Sanjuan, Víctor Manuel Expósito-Suárez, Cristina Argiz, Pedro Mora, Joseph Emmanuel Ndjana Nkoulou, Marta Barragán, José Francisco Benavente

**Affiliations:** 1 Environmental Radioactivity and Radiological Surveillance Unit, Department for Environment, CIEMAT (Research Center of Energy, Environmental and Technology), Av. Complutense, 22, 28040 Madrid, Spain; victormanuel.exposito@ciemat.es (V.M.E.-S.); marta.barragan@ciemat.es (M.B.); jf.benavente@ciemat.es (J.F.B.); 2 Department of Building Materials, Civil Engineering School, Technical University of Madrid, C/Profesor Aranguren, s/n, Ciudad Universitaria, 28040 Madrid, Spain; masanjuan@ieca.es; 3 Civil Engineering School, Technical University of Madrid, C/Profesor Aranguren, 3, Ciudad Universitaria, 28040 Madrid, Spain; cg.argiz@upm.es; 4 Department of Geological and Mines Engineering, Mine and Energy Engineering School, Technical University of Madrid, C/Ríos Rosas, 21, 28003 Madrid, Spain; pmora@oficemen.com; 5 Research Centre for Nuclear Science and Technology, Institute of Geological and Mining Research, Yaoundé P.O. BOX 4110, Cameroon; nndjana@yahoo.fr

**Keywords:** NORM materials, activity concentration index, limestone, GGBFS, recycled concrete fines

## Abstract

**Highlights:**

- Eight Portland cement types with L, F, S additions assessed radiologically.
- ^226^Ra, ^232^Th, ^40^K measured via HPGe spectrometry and radiochemical methods.
- ^222^Rn emanation quantified by accumulation method in cements and mortars.
- RESRAD-BUILD estimated annual effective doses in a 35 m^2^ dwelling model.
- All cements meet radiological safety criteria for circular-economy use.

**Main findings**

- Slag activity reaches 113 Bq kg^−1^ ^238^U, highest among individual additions.
- S-containing cements (CEM II/C-M, VI(S-L), VI(S-F)) exceed 50 Bq kg^−1^ average.
- All cured mortars remain below the 50 Bq kg^−1^ building-material average.
- ^222^Rn inhalation, not external gamma, dominates total effective dose.
- Slag shows lowest radon emanation fraction 0.0607 ± 0.0034, among additions.

**Implications of the main findings**

- Findings confirm EN 197-6 additions are radiologically safe for use.
- Slag dosage should be verified to control radionuclide enrichment.
- RESRAD-BUILD confirms 0.32 mSv infant dose is within safety limits.
- Results support circular-economy reuse of CDW fines in cement.
- Low slag radon emanation favors its use to limit indoor ^222^Rn exposure.

**Abstract:**

The incorporation of recycled concrete fines (F), limestone (L), and ground granulated blast-furnace slag (S) as Portland cement constituents in accordance with EN 197-6 requires the determination of naturally occurring radionuclides to ensure radiological safety from a radiation protection standpoint. This study carried out a radiological assessment of eight cement types with varying proportions of L, F, and S additions, including anhydrous cements and mortars cured for 2 and 28 days. The activity concentrations of ^226^Ra, ^232^Th, and ^40^K were determined by gamma-ray spectrometry using HPGe detectors and radiochemical separation. In addition, the ^222^Rn emanation fractions were measured by the accumulation method using an AlphaGuard detector. Finally, annual effective doses were calculated using the RESRAD-BUILD software for a standard dwelling of 35 m^2^ occupied by an adult and an infant. Among the individual materials, S exhibited the highest activity in the uranium decay series (113 ± 24 Bq kg^−1^ of ^238^U), while L and F showed comparably lower values. Cements containing S additions (CEM II/C-M, CEM VI (S-L), and CEM VI (S-F)) were higher than the reference average value for building materials of 50 Bq kg^−1^, although all mortars remained below this value. Principal component analysis revealed significant correlations between S content, SiO_2_, Al_2_O_3_, and ^232^Th. The maximum annual effective dose reached 0.32 mSv for infants (CEM VI (S-L)), with the dose due to ^222^Rn inhalation being predominant. All cements studied are radiologically safe, confirming their suitability for use within the objectives of the circular economy.

## 1. Introduction

In 2023, the European Technical Committee for Standardization CEN/TC 51 “Cement and building lime” prepared the European standard EN 197-6 “Cement—Part 6: Cement with recycled building materials” [1]. This is a good example of climate change mitigation through the circular economy; i.e., the circular economy can reduce carbon dioxide emissions in Europe. It also reveals the socio-economic and environmental co-benefits that circular mitigation options can bring to EU countries. This study includes recycled concrete fines as a new supplementary material added to cements, in addition to those already considered previously, such as limestone and ground granulated blast-furnace slag. In this way, the aim is to consider three additions both individually and in combination, in order to assess their use from a radiation protection standpoint.

Construction and Demolition Waste (CDW) represents the largest waste stream in the European Union, amounting to approximately 850 million tons each year (around 36–40% of all waste generated). In response, the EU has established a 70% recovery target under the Waste Framework Directive and the Circular Economy Action Plan [2]. These policies seek to strengthen confidence in recycled materials, advance selective demolition practices, and encourage on-site sorting as essential steps toward a more circular, resource-efficient built environment [3].

The construction sector constitutes the most immediate and strategically significant end-use pathway for construction and demolition waste (CDW), owing to its exceptional capacity to reabsorb substantial volumes of mineral-based materials. Its principal applications include the production of recycled aggregates for road infrastructure and civil engineering works, reclaimed asphalt materials, recycled aggregate concrete, backfilling operations, and non-structural construction products such as masonry blocks, paving units, and precast elements [4], and now, part of them as a new Portland Cement constituent [1].

Recycled concrete fines may be regarded as a specific case of CDWs and are defined in the EU standard as specially selected and prepared mineral materials [1], which are (i) coming from plants or units producing recycled concrete aggregates and/or sands; (ii) reclaimed from concrete production operations; (iii) coming from reclaimed crushed aggregates as defined in EN 206:2013+A2:2021 [5].

The potential radiological risk of Portland cements strongly depends on the nature and origin of their constituents other than Portland clinker [6]. In general, all mineral-based construction materials contain naturally occurring radionuclides (NORM), mainly from the uranium and thorium decay series and ^40^K; therefore, radiological assessment is commonly based on the activity concentrations of ^226^Ra, ^232^Th, and ^40^K. This approach falls within the international concern regarding building materials containing naturally occurring radionuclides of non-artificial origin above natural background radiation levels (NORM), since these materials constitute one of the main sources of public exposure to natural radiation indoors, both from external gamma radiation and from ^222^Rn inhalation, as reflected in the reference framework established by UNSCEAR [7]. In blended cements, limestone (L, LL) usually presents the lowest radiological concern [8], as limestone often dilutes the radionuclide content of the clinker fraction. Ground granulated blast-furnace slag (GGBFS) generally shows low to moderate risk, although its radiological performance depends on the industrial process and raw materials involved [9]. Fly ash may represent a comparatively higher potential risk because coal combustion can concentrate naturally occurring radionuclides in the ash fraction [10], making source-specific characterization essential. Natural pozzolans and calcined clays show variable performance, as their radionuclide content is controlled by local geology; volcanic materials, in particular, may contain elevated levels of ^40^K or thorium-bearing minerals. Silica fume typically contributes limited radiological impact due to its low dosage in cement formulations, but it should still be verified analytically. Overall, the radiological safety of blended Portland cements cannot be generalized. Therefore, it must be assessed through measured radionuclide activity concentrations by type of cement constituent and relevant dose before large-scale use in construction. Recycled concrete fines [1] may also present heterogeneous radiological profiles, depending on the parent materials from which they originate. Although previous studies have addressed the radiological characteristics of conventional cement additions, such as ground granulated blast-furnace slag [8,9], fly ash [10], and natural pozzolans, the radiological assessment of recycled concrete fines as a standardized cement constituent remains scarce in the literature, given that this addition was only formally incorporated into European standards following the publication of EN 197-6 in 2023 [1]. Consequently, no systematic data are currently available to compare the radiological behavior of recycled concrete fines, either individually or in combination with limestone and slag, across the different stages of the cement life cycle (individual material, anhydrous cement, and hardened mortar). This study therefore provides a novel dataset that integrates the determination of natural gamma-emitting radionuclides, ^222^Rn emanation, and the estimation of annual effective dose rates using the RESRAD-BUILD code. The relationship between chemical composition and radiological content was assessed by means of principal component analysis. This multidisciplinary approach represents a contribution with respect to previous existing studies, which were generally focused on a single type of addition or on a single stage of the cement manufacturing process.

The aim of this work is to ensure the safe use of three different additives from a radiological protection standpoint, along with their valorization from the same perspective. To this end, seven types of cement were selected, incorporating different additives of limestone (L), recycled concrete fines (F), and granulated blast-furnace slag (S). The specific objectives established to achieve this aim were: (i) to determine the activity concentration of natural gamma emitters and the ^222^Rn exhalation rate of the individual materials (limestone (L), demolition fines (F), and granulated blast-furnace slag (S)) by means of gamma-ray spectrometry using semiconductor detectors and the AlphaGuard detector; (ii) to determine the activity concentration of gamma emitters and the ^222^Rn emanation of anhydrous cements containing L+F+S additives, correlating their chemical composition with the corresponding radioactive content; and (iii) to characterize the mortars manufactured from these cements using standardized sand, studying ^222^Rn emanation and determining the annual effective dose for a typical dwelling using the RESRAD-Build code.

## 2. Materials and Methods

### 2.1. Materials and Chemical Composition

Table 1 shows the percentage composition of the different cements studied in this work. The CEM I sample represents a reference cement, as it contains no additions of limestone (L), recycled concrete fines (F), or granulated blast-furnace slag (S). The cements containing only a single addition were CEM II/A-L and CEM II/B-L, with 20% and 35% limestone, respectively, and CEM II/A-F and CEM II/B-F, with 20% and 35% recycled concrete fines, respectively. The remaining cements contained several additions, as shown in Table 1 (CEM II/C-M, CEM VI (S-L), and CEM VI (S-F)). The compositions of the cements studied do not correspond to specific commercial products sampled directly at a production plant. These cements were formulated in the laboratory by combining Portland clinker with individual additions of limestone, recycled concrete fines, and ground granulated blast-furnace slag, using the maximum proportions established by the EN 197-6 standard for each cement type and subtype (Table 1). This experimental design allows the radiological effect of each addition to be systematically assessed, both individually and in combination, thereby ensuring that the results obtained are applicable to the future commercial production of these cements in accordance with EN 197-6.

The mortars prepared with the cements listed in Table 1 were made using standardized sand. The radioactive content of the standardized sand is very low and can be considered negligible in comparison with the other materials used to prepare these mortars [11,12]. The recycled concrete fines (F) used in this study were supplied by a construction and demolition waste recycling plant, complying with the requirements established in the EN 197-6 standard, according to which the starting recycled aggregate must contain a minimum of 90% old concrete, thereby ensuring a relatively homogeneous mineralogical composition, mainly comprising quartz, calcite, and hydrated cement paste. Nevertheless, it is acknowledged that the compositional variability of construction and demolition waste constitutes a relevant factor to be considered in its valorization as a supplementary cementitious material, as recently described in studies on commercial mixed recycled aggregates [13]. Therefore, the radiological results obtained in this study should be interpreted in relation to the specific provenance of the material used, and should not be directly extrapolated to recycled concrete fines of a different geographical or industrial origin.

The chemical composition of the samples was determined by X-ray fluorescence using a Philips PW-1404 sequential spectrometer in accordance with standard UNE-EN 15410:2012 [14]. The equipment was fitted with an X-ray tube with a radium anode, operating at a voltage between 30 and 50 kV and a current between 0.4 and 0.8 mA under vacuum conditions. The XRF spectra were obtained using a counting time of between 60 and 180 s for each sample. Loss on ignition was determined in accordance with European standard EN 196-2 [15]. The equipment software enabled the quantification, expressed as oxides, of Al_2_O_3_, CaO, Fe_2_O_3_, K_2_O, MgO, Na_2_O, SO_3_, SiO_2_, and TiO_2_ present in the analyzed cement samples. The samples were measured only once, as the laboratory was accredited to ISO/IEC 17025:2017 [16], which guarantees the reproducibility of the results.

### 2.2. Gamma-Ray Spectrometry

The gamma-emitting radionuclides present in the samples analyzed in this study were determined by gamma-ray spectrometry using HPGe detectors. The detectors used were of the extended-range type (XtRa), with relative efficiencies compared to a NaI(Tl) detector of 42.1% and 115.7%, respectively. The detectors were fitted with passive lead shielding 15 cm thick, with two additional layers of Cu and Zn, 2.0 mm and 1.0 mm thick, respectively. The detectors were controlled by integrated DSA-LX modules, which contain all the necessary associated electronics: a high-voltage power supply, an amplifier, an analog-to-digital converter, and a peak stabilizer [17]. The modules were connected to a PC on which Genie 2000 software was installed, with which the spectra were acquired and analyzed. Energy calibration as a function of channel number was carried out using point sources supplied by the Ionizing Radiation Metrology Laboratory of CIEMAT (LMRI), containing the following radionuclides and energies [18]: ^210^Pb (46.539 (1) keV), ^241^Am (59.5409 (1) keV), ^137^Cs (661.657 (3) keV), ^88^Y (898.042 (11) keV and 1836.070 (8) keV), and ^60^Co (1173.228 (3) keV and 1332.492 (4) keV). Efficiency calibration as a function of energy was performed using LabSOCS software (Geometry Composer, Version 4.4.1, 2016), as both detectors used were characterized by Mirion-Canberra [19]. Likewise, the true coincidence summing correction was carried out using the Peak-To-Total method in Genie 2000 [20]. The geometry used to measure the samples consisted of a cylindrical polypropylene box 76 mm in diameter and 30 mm in height, whose counting efficiency was validated in a previous study [21]. Furthermore, the spectral interferences caused by ^235^U on the 186.211 (13) keV photopeak of ^226^Ra and by ^228^Ac on the 1460.851 (6) keV photopeak of ^40^K were suppressed using the method proposed in a previous study [22]. The samples were measured for a minimum of 80,000 s, and internal quality procedures were followed for background measurements, calibration frequency, and quality controls, as the laboratory was accredited under ISO/IEC 17025:2017 [16].

### 2.3. Radiochemical Methods

The calculation of the effective doses performed in this work required the determination of ^226^Ra, ^232^Th, and ^40^K. ^40^K was determined by gamma-ray spectrometry (Section 2.2), whereas ^226^Ra and ^232^Th were determined by radiochemical separation, since the activity concentrations expected in the cement samples were of the order of 50 Bq kg^−1^ and therefore exhibited a high measurement uncertainty when determined by direct gamma-ray measurement [11].

#### 2.3.1. Radiochemical Separation Method for ^232^Th

^232^Th was determined using the radiochemical method developed in [23]. Briefly, the radiochemical method consists of the liquid–liquid extraction of Th from a 4 M HCl solution. The organic phase obtained was purified of U by means of a second liquid–liquid extraction with H_2_O, and the Th present in the aqueous phase was recovered by precipitation with Fe(OH)_3_. The Fe(OH)_3_ precipitate was then dissolved with 10 mL of 8 M HNO_3_, and the Th was extracted into the organic phase once again. Finally, the Th was back-extracted from the organic phase with 4 M HCl and electrodeposited onto a stainless-steel planchet using the Hallstadius method [24] for 2 h at a current of 1 A. The samples were measured using an AlphaAnalyst system fitted with PIPS semiconductor detectors for 500,000 s, with the same counting time used for background measurements. The spectra were acquired and analyzed using Genie 2000 software. Although this method was not accredited, the same procedures as those followed for gamma-ray spectrometry were applied with regard to quality controls, calibration and participation in intercomparison exercises. The uncertainty, decision threshold and detection limit were determined in accordance with ISO 11929 [25], the average detection limit being 2.6 Bq kg^−1^ for a counting time of 500,000 s and an aliquot of 0.5 g.

#### 2.3.2. Radiochemical Separation Method for ^226^Ra

The radiochemical separation method for ^226^Ra was carried out following the method proposed in [26]. Briefly, the method consisted of a first stage based on the Goldin method [27], yielding a purified [BaRa]SO_4_ precipitate free of alpha emitters (Th, U, Po, etc.). The precipitate was converted into [BaRa]CO_3_ by dissolving the [BaRa]SO_4_ with a saturated H_2_CO_3_ solution at 90 °C. The [BaRa]CO_3_ precipitate was dissolved with a 3 M HNO_3_ solution and precipitated as [BaRa]Cr_2_O_7_ at pH 4.2. The [BaRa]Cr_2_O_7_ was then converted into [BaRa]Cl_2_ using a 1:6 solution of diethyl ether and HCl. Finally, the [BaRa]Cl_2_ was dissolved in H_2_O and precipitated once again as [BaRa]SO_4_ using a 1 M H_2_SO_4_ solution. Once purified of stable cations, the precipitate was dried and measured using a ZnS(Ag) solid scintillation counter for 80,000 s. The background counting time was 200,000 s. The activity of ^226^Ra was calculated using the Bateman equations to correct for the growth of ^222^Rn, ^218^Po and ^214^Po, and the decay of the ^224^Ra and ^223^Ra present [28]. As with the radiochemical method for ^232^Th, this method was not accredited; however, the same quality controls, calibrations and participation in intercomparison exercises were followed as for gamma-ray spectrometry. The uncertainty, decision threshold and detection limit were determined in accordance with ISO 11929 [25], the latter being 1.7 Bq kg^−1^ for a counting time of 200,000 s and an aliquot of 0.5 g.

### 2.4. Determination of the Emanation Fraction

The emanation fraction was determined using the accumulation method developed in a previous study [29]. The method consists of first obtaining the exhalation coefficient (ERn222, Bq kg^−1^ s^−1^) from the equilibrium ^222^Rn concentration ($C_{eq}$, Bq m^−3^):

(1) ERn222=Ceq⋅V⋅λeffm

where *V* is the volume of the accumulation chamber (m^3^), *λ_eff_* is the effective decay constant of ^222^Rn, and *m* is the mass of the sample aliquot measured, in kg. The amount of sample analyzed was less than 1/10 of the volume of the accumulation chamber, in order to prevent reabsorption of ^222^Rn into the sample. The emanation fraction (F_e_) is given by expression (2):

(2) Fe=ERn222ARa226⋅λRn222

where ARa226 is the activity concentration of ^226^Ra of the aliquot measured (Bq kg^−1^) and λRn222 (s^−1^). The uncertainty, decision threshold, and detection limits were all determined based on the criteria set out in standard ISO 11929 [25], which were applied following the approach developed in a previous study [29].

### 2.5. Determination of Effective Doses in a Reference Dwelling Using RESRAD

**Figure 1:**
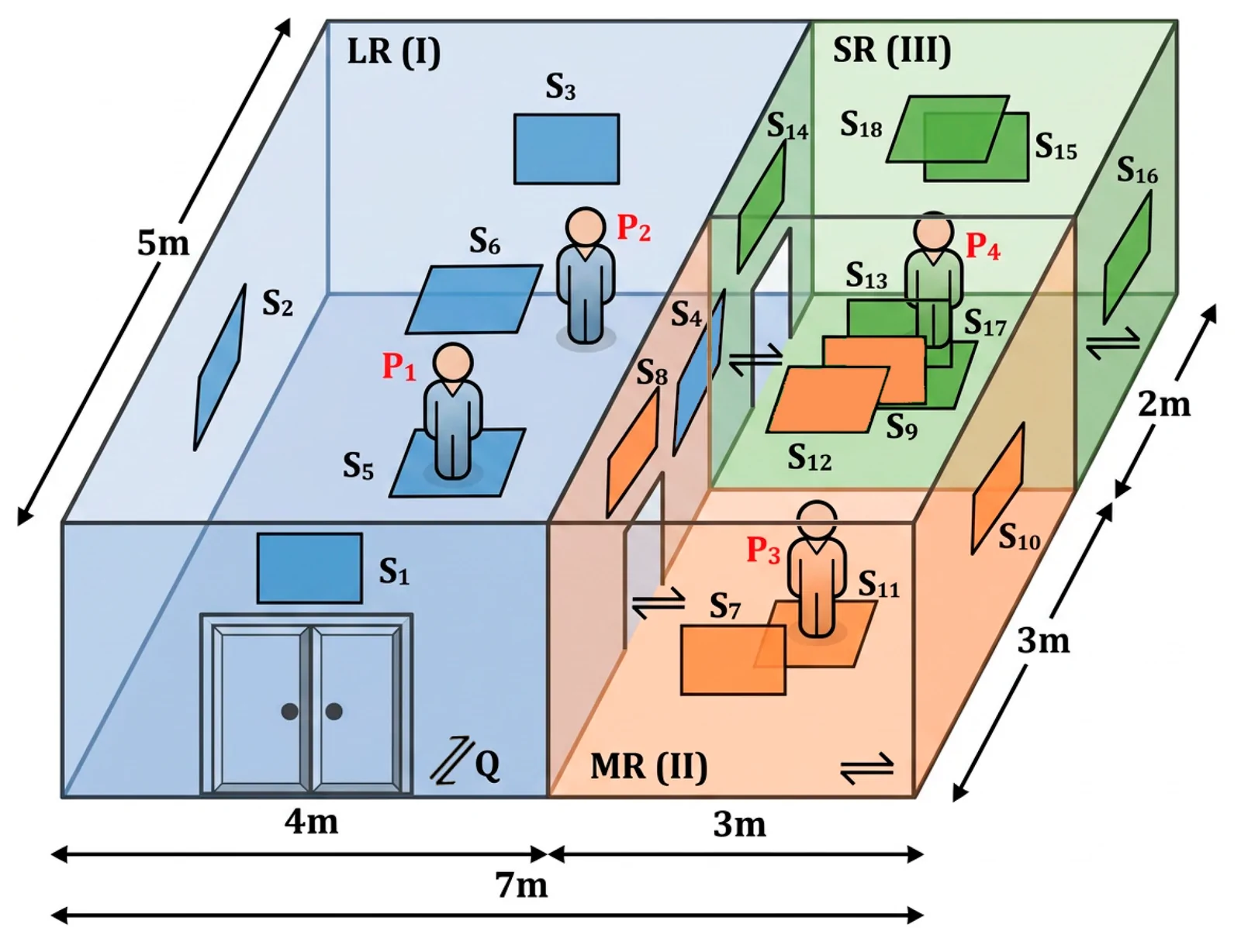
Schematic diagram of the reference dwelling with an area of 35 m^2^, comprising three types of rooms: (1) a living room measuring 4 × 5 × 2.8 m^3^ (blue color), (2) a main bedroom measuring 3 × 3 × 2.8 m^3^ (orange color), and (3) a secondary bedroom measuring 3 × 2 × 2.8 m^3^ (green color), used to calculate the annual effective dose rates.

The annual effective dose rate was determined for a reference dwelling with an area of 35 m^2^, comprising three types of rooms: (1) a living room measuring 4 × 5 × 2.8 m^3^, (2) a main bedroom measuring 3 × 3 × 2.8 m, and (3) a secondary bedroom measuring 3 × 2 × 2.8 m^3^ (Figure 1). The occupants of this dwelling are an adult and an infant. The doses were determined using the RESRAD-BUILD 3.5. software (Residual Radioactive Material Guidelines for Buildings), a computer code developed by Argonne National Laboratory for the U.S. Department of Energy and the U.S. Nuclear Regulatory Commission to assess the radiation doses and radiological risks to which people living or working in buildings containing radioactive materials are exposed. The dimensions considered were similar to those included in European Directive 2013/59/Euratom [30]. The input variables used to obtain the annual effective dose rate due to external radiation were the activity concentrations of ^226^Ra, ^232^Th, and ^40^K. The doses were integrated over 18 different positions, as shown in Figure 1. Furthermore, unlike the dose estimation method set out in Directive 2013/59/Euratom, the RESRAD-BUILD software also allows for the determination of the effective inhalation dose due to the activity concentration of ^222^Rn. For this reason, the value of the ^222^Rn emanation fraction (Section 2.4) was introduced into the model to assess this contribution to the annual effective dose rate. Therefore, the annual effective dose rate obtained using the RESRAD-BUILD software represents the total dose, combining both external radiation and that resulting from the inhalation of ^222^Rn.

### 2.6. Statistical Analysis

The comparison between the activity concentration of natural radionuclides from the natural radioactive series, grouped as ^226^Ra and ^232^Th, and ^40^K, and the percentage of oxides obtained by X-ray fluorescence (XRF) was performed using principal component analysis (PCA). Dimensionality reduction of the final variables was achieved by removing redundant and uninformative variables. Identification and reduction of the variables were conducted through a four-stage process. The initial set of variables subjected to the dimensionality reduction process of the PCA included the activity concentrations of ^226^Ra, ^232^Th, and ^40^K, along with the oxide percentages determined by XRF (Al_2_O_3_, CaO, Fe_2_O_3_, K_2_O, MgO, Na_2_O, SO_3_, SiO_2_, TiO_2_, P_2_O_5_, and Cl). The activity concentrations of ^226^Ra, ^232^Th, and ^40^K were selected from the set of radioactive variables since they are the main radionuclides of the uranium and thorium series, and ^40^K is the most abundant natural radionuclide in the Earth’s crust. The first stage excluded variables with a communality lower than 0.5. The second stage removed variables with factor loadings below 0.10 in all components. The third stage discarded variables with a VIF higher than 10.0, indicative of extreme multicollinearity. Finally, in the fourth stage, the PCA was repeated with the remaining variables, iteratively removing those with a communality/loading ratio below 0.2 and 0.10, respectively, until the final set of six variables was obtained.

## 3. Results and Discussion

### 3.1. Individual Materials

The materials added to the cements studied in this work were: limestone (L), granulated blast-furnace slag (S), and demolition fines (F). Table 2 shows the activity concentrations of the natural radionuclides belonging to the natural uranium and thorium radioactive series, together with ^40^K. The activity concentration of ^226^Ra was determined by gamma-ray spectrometry (Section 2.2) and by radiochemical separation (Section 2.3.2). This dual determination was carried out to confirm the radioactive equilibrium between ^226^Ra/^214^Pb, since the determination of ^226^Ra by gamma-ray spectrometry is subject to high uncertainty due to the correction required for the interference of ^235^U on the 186.211 (13) keV photopeak [22]. The samples composed of L and S had a similar radioactive content in both the uranium and thorium series, being slightly higher in the case of F. However, the radionuclides of the natural uranium radioactive series (^234^Th, ^226^Ra, ^214^Pb, ^214^Bi, and ^210^Pb) contained in the S sample were higher than the average of 50 Bq kg^−1^ for construction materials [31]. On the other hand, the radionuclides of the thorium series (^228^Ac, ^212^Pb, and ^40^K) showed values very close to the worldwide average for construction materials (50 Bq kg^−1^). The level of 50 Bq kg^−1^, used for the uranium and thorium series, corresponds to the average activity concentration reported for building materials in the European database [31] and does not constitute either a regulatory limit or an exemption threshold. Therefore, this value should be regarded as a reference point for contextualizing the differences found between the building materials used in this study. The activity concentrations of the standard sand for the natural uranium and thorium radioactive series were much lower than those of the remaining samples. Finally, ^40^K was well below the 500 Bq kg^−1^ world average value for construction materials, with the standard sand showing the highest value due to the presence of potassium feldspar [32]. Therefore, S would be the material that could contribute the most to the effective dose from external radiation. Regarding the secular equilibrium of the uranium and thorium series in these three materials, it was observed that, in general, the radionuclides of both series were in secular equilibrium. However, the S sample showed a disequilibrium between ^210^Pb and the remaining members of the natural uranium radioactive series. This disequilibrium is due to the fact that the temperature reached during blast furnace formation is around 1600 °C, which is very close to the boiling point of ^210^Pb of 1740 °C [33]. Furthermore, the difference observed between the activity concentrations of ^228^Ac and ^212^Pb with respect to ^208^Tl is due to the branching ratio in the decay of ^212^Bi, which is 36% [34].

The emanation fractions of the L and F samples were 0.418 ± 0.044 and 0.451 ± 0.047, respectively. However, the emanation fraction of the S sample was 0.0607 ± 0.0034. The emanation fractions found for limestones were equivalent to those obtained for the limestone studied in this work [35]. On the other hand, the activity concentrations of the F sample are consistent with studies analyzing different concretes, where the range varied between 1.0 and 85% [36]. Furthermore, Sas et al. [37] found that pure granulated blast-furnace slags (GGBFS) exhibited very low emanation factors (0.6 ± 0.1%), which resulted in emanation values of 6.0 ± 0.9% in Alkali-Activated Materials (AAM) mixtures with a high GGBFS content, demonstrating the beneficial effect of this material in reducing radon exhalation. These results are consistent with those obtained in the present study. The slag (S) showed the lowest ^222^Rn emanation fraction despite having the highest ^226^Ra activity concentration compared with the other materials studied. This fact could be attributed to the rapid cooling of the molten material that occurs during the formation of granulated slag in the blast furnace. This rapid cooling would give rise to a dense and homogeneous aluminosilicate network in which the radionuclides would become structurally incorporated, rather than being preferentially located in crystalline phases or on the surface of the grains [38]. The formation of this crystalline network would restrict the position of ^226^Ra within the open porous network, thereby reducing the migration of ^222^Rn towards the outside of the grain. Nevertheless, this interpretation should be regarded as a mechanistic hypothesis consistent with the observed data, rather than as a mechanism experimentally confirmed in this study, and its verification by means of spectroscopic techniques (e.g., Raman or NMR) could be addressed in future studies.

### 3.2. Cements and Mortars

**Figure 2:**
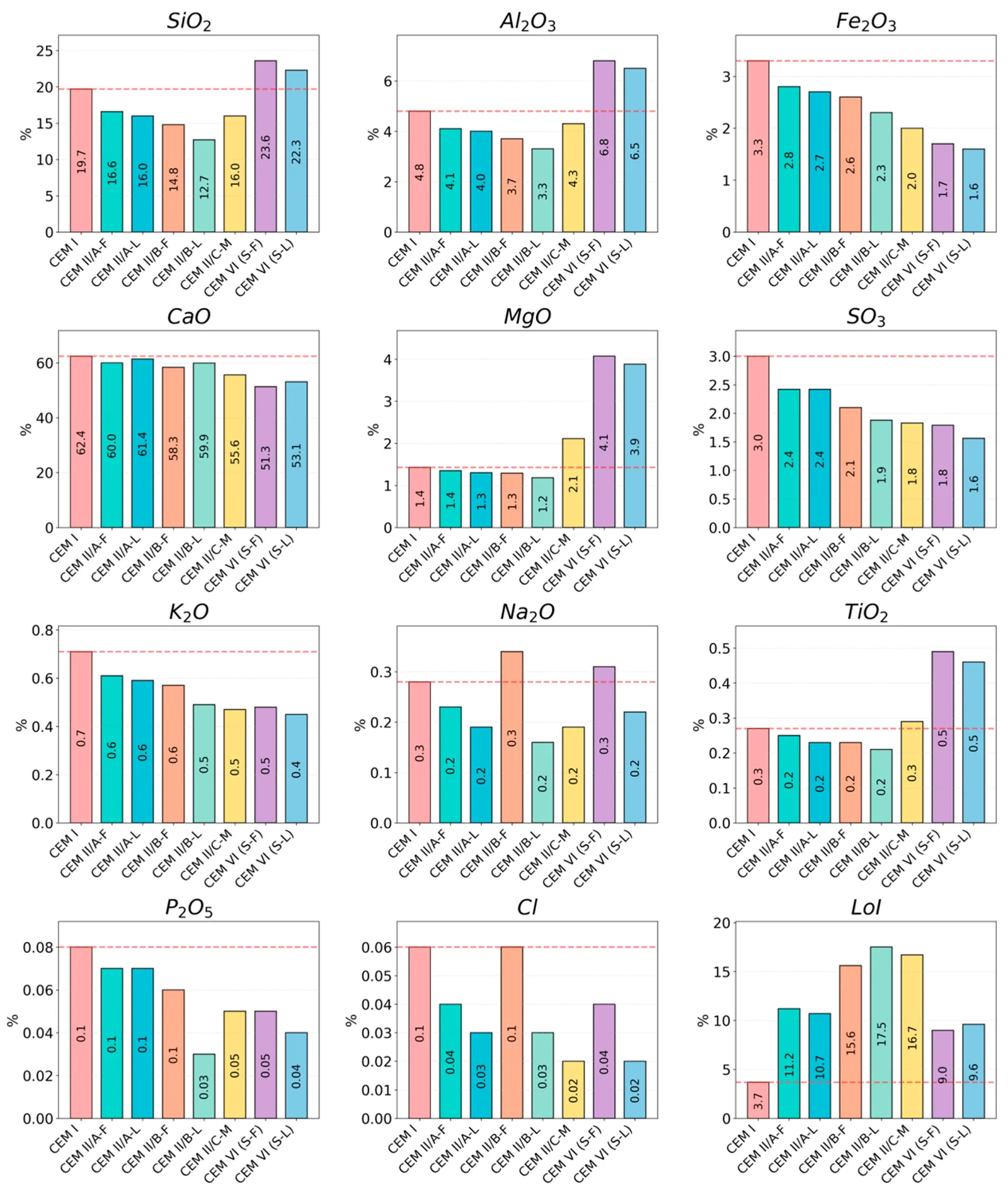
Percentages of SiO_2_, Al_2_O_3_, Fe_2_O_3_, CaO, MgO, SO_3_, K_2_O, Na_2_O, TiO_2_, P_2_O_5_ and Cl determined by X-ray fluorescence. The loss on ignition was determined in accordance with the EN 196-2 standard. The dashed line represents the CEM I value used as the reference value.

Figure 2 shows the oxide ratios of the different anhydrous cements analyzed by X-ray fluorescence (XRF). The results showed that the SiO_2_, Al_2_O_3_, MgO, and TiO_2_ oxides were higher relative to the type I cement (CEM I) in the three cements containing S, namely CEM II/C-M (15%), CEM VI (S-L) (45%), and CEM VI (S-F) (45%). The increase observed in the SiO_2_, Al_2_O_3_, MgO, and TiO_2_ oxides was also reported by Jamil et al. [39] when adding GGBFS as an additive in the formation of geopolymers manufactured with kaolin equivalent to those tested in this study. The increase in these oxides is due to the fact that GGBFS exhibits variable compositions of the CaO-MgO-Al_2_O_3_-SiO_2_ system depending on the composition of the iron minerals, coke, and coal used in the furnace, as well as the CaO and MgO used as fluxes [40]. The oxides remained constant or decreased, reflecting that CEM I was the source contributing that oxide, as observed for Fe_2_O_3_, SO_3_, K_2_O, and P_2_O_5_. On the other hand, the cements containing demolition concrete fines showed an increase in Na_2_O and Cl, which could be attributed either to possible additives in the concrete or to the aggregates used [41]. However, the increase in Na_2_O and Cl had a lower weight in the composition than in the case of GGBFS (SiO_2_, Al_2_O_3_, MgO, and TiO_2_).

Table 3 contains the activity concentrations of the natural radionuclides belonging to the natural uranium radioactive series (^234^Th, ^226^Ra, ^214^Pb, ^214^Bi, and ^210^Pb) and thorium series (^232^Th, ^228^Ac, ^212^Pb, and ^208^Tl), together with the activity concentration of ^40^K for the anhydrous cements and the mortars prepared from these cements. The radioactive content of the mortars was determined at 2 and 28 days of curing. The activity concentrations were higher for the anhydrous cements than for the mortars. The largest contribution to the radioactive content was due to the cement, and in the case of the mortars, this content was diluted by the 66% proportion of siliceous aggregate and the hydration water used to prepare the mortars. The cements with the highest radioactive content were those containing ground granulated blast-furnace slag (GGBFS): CEM II/C-M, CEM VI (S-L), and CEM VI (S-F). These results are consistent with those shown in Table 2, as GGBFS was the material with the highest radioactive content (113 ± 24 Bq kg^−1^ of ^238^U in secular equilibrium). This content in GGBFS meant that the CEM VI (S-L) and CEM VI (S-F) cements exceeded the average of 50 Bq kg^−1^ typical of construction materials [31]. On the other hand, the activity concentration of ^40^K was uniform across all cements, with a mean of 116.2 ± 5.4 Bq kg^−1^, in both the anhydrous cements and the mortars. Therefore, although the siliceous aggregate does not contribute any radioactive content, it does not offset its dilution effect within the mortar percentage. Likewise, the siliceous aggregate is responsible for all the mortars having nearly the same activity concentration of ^40^K. This behavior can be explained by the geochemical partitioning mechanism that occurs during slag formation in the blast furnace. During the smelting process, impurities in the iron ore (mainly silica and alumina) separate from the molten metal and become concentrated in the slag phase, while the metallic iron is recovered separately [6]. Moreover, Th is a lithophile element with low solubility in the metallic phase. For this reason, Th tends to partition into the silica of the slag along with Al_2_O_3_ and SiO_2_. Therefore, this behavior would explain the correlation observed between ^232^Th, Al_2_O_3_, and SiO_2_ in the principal component analysis. Furthermore, the degree of enrichment in natural radionuclides in the slag depends on both the geological origin of the iron ore and the type of industrial process employed, such as the type of furnace in which these materials are produced [6].

**Figure 3:**
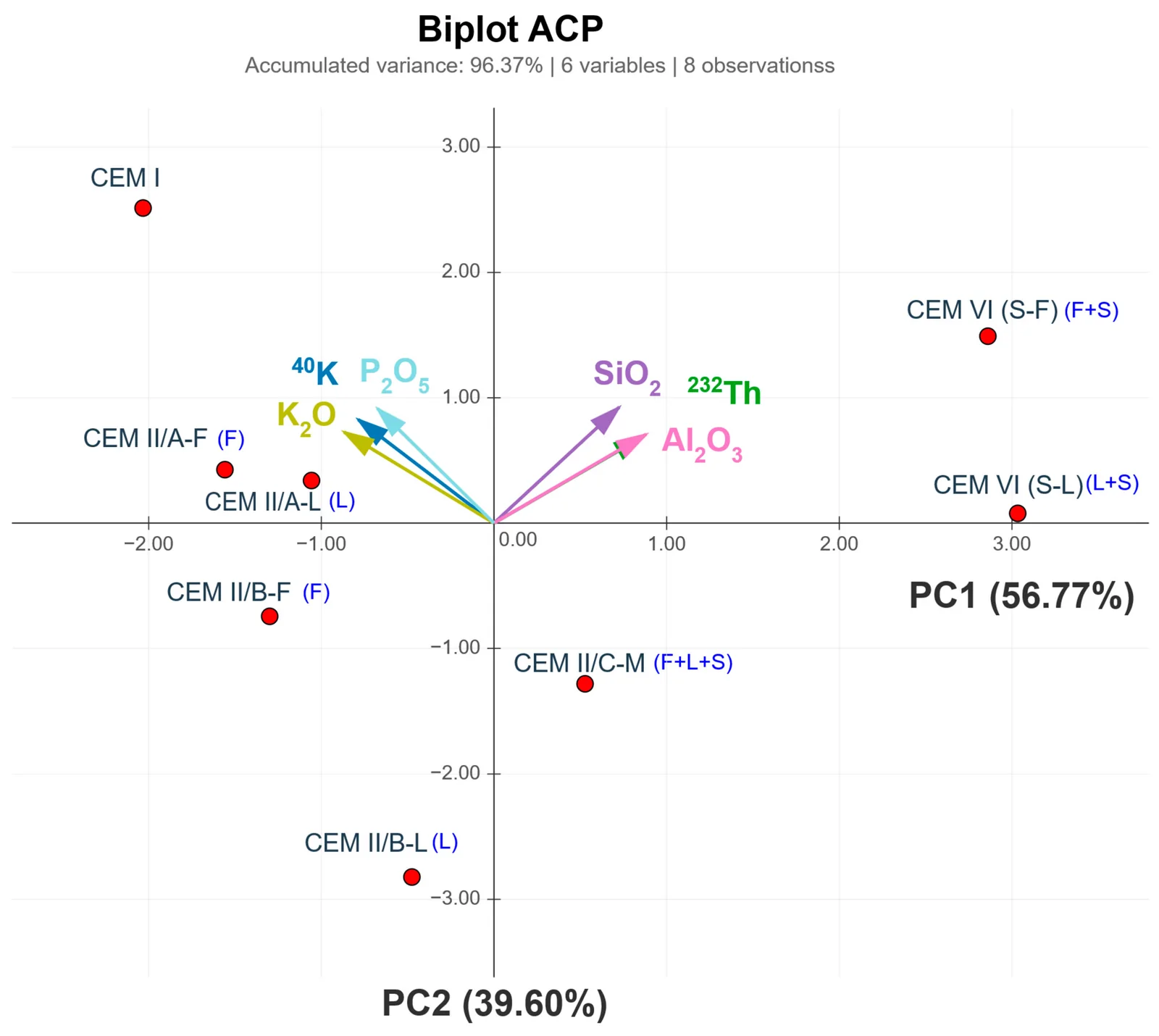
HJ-Biplot plot resulting from the principal component analysis between the chemical composition obtained by X-ray fluorescence and the activity concentrations of ^226^Ra, ^232^Th, and ^40^K. The composition of each of the cements is detailed in blue in the graph.

Figure 3 shows the HJ-Biplot resulting from the principal component analysis (PCA) between the chemical composition obtained by X-ray fluorescence and the activity concentrations of ^226^Ra, ^232^Th, and ^40^K. The variables resulting from the iterative elimination process described in Section 2.6 were, from left to right: K_2_O, ^40^K, P_2_O_5_, SiO_2_, ^232^Th, and Al_2_O_3_, such that the PCA condition requiring the number of variables to be lower than the number of observations was satisfied. The PCA accounted for 96.37% of the cumulative variance, distributed as 56.77% for component 1 and 39.60% for component 2. The weight exerted by the different variables on the two components was greater for component 1 than for component 2. Therefore, the HJ-Biplot plot should be interpreted with reference to component 1. In this respect, the cements can be distinguished between those whose score places them to the left of the *X*-axis, namely CEM I, CEM II/A-F, CEM II/A-L, CEM II/B-F, and CEM II/B-L, and those that fall to the right of the *Y*-axis: CEM VI (S-F), CEM VI (S-L), and CEM II/C-M. This observed differentiation is consistent with the composition of the cements (Table 1), since cements with a clinker content above 65% are distinguished from those with a lower clinker content. Likewise, cements with a clinker percentage equal to or lower than 50% and containing blast-furnace slag (GGBFS) were positioned to the right of the *Y*-axis. Therefore, the cements with a higher clinker percentage are characterized by higher ^40^K activity concentrations and higher percentages of K_2_O and P_2_O_5_ oxides. ^40^K and K_2_O showed a high correlation with each other, which is logical since ^40^K is an isotope of stable K with a fixed isotopic abundance of 0.0117% [18]. The contribution of ^40^K was higher in CEM I, which contains between 95 and 100% clinker, and therefore in those cements with a higher clinker percentage, which, as indicated above, are those positioned to the left of the *Y*-axis in the biplot. Likewise, the greater presence of P_2_O_5_ in CEM I, and therefore in cements with a higher clinker percentage, is due to phosphorus being structurally incorporated into the silicate-bearing clinker phases, such as belite (C_2_S), as it condenses into this phase in the kiln [42]. This incorporation is due to the possible use of fuels, which could be mainly attributed to the use of secondary fuels such as meat and bone meal, which contain Ca_3_(PO_4_)_2_ [43]. On the other hand, the greater presence of SiO_2_ and Al_2_O_3_ in the cements containing granulated blast-furnace slag (GGBFS) (CEM II/C-M, CEM VI (S-L), and CEM VI (S-F)) is due to the fact that silica and calcined bauxite are used as fluxes in the GGBFS formation process. Likewise, Lewicka et al. also found that cements incorporating GGBFS had a greater presence of ^232^Th [44].

Table 4 shows the emanation fractions for the individual anhydrous cements and for the mortars prepared at curing ages of 2 and 28 days. The results showed that the cements with limestone addition (CEM II/A-L and CEM II/B-L) increased progressively from the anhydrous cement to the mortars aged 2 and 28 days. Although limestone addition of up to a maximum of 30% causes a reduction in the pores of the final mortar, this suggests, as a plausible hypothesis rather than a confirmed mechanism, that the carboaluminate formation reactions occurring in these cements cause a repositioning of ^226^Ra within the crystal structure that leads to greater emanation [45]. This effect was already considered in a previous study [29]. On the other hand, the cements with recycled concrete fines addition (F) showed stable behavior, with no variation observed in ^222^Rn emanation. The cements containing granulated blast-furnace slag showed different behavior, since the ^222^Rn emanation generally increased from the anhydrous cement to the 2-day-old mortar, but subsequently decreased in the cements containing limestone (CEM II/C-M and CEM VI (S-L)), whereas it increased in the cement containing concrete fines and slag (CEM VI (S-F)). This behavior could be attributed to structural changes caused by the hydration reactions, which would lead to changes in the position of ^226^Ra within the crystal structure.

In addition to the repositioning of ^226^Ra within the crystal lattice, the evolution of the emanation fraction between anhydrous cement and hydrated mortars can be explained by considering the transport mechanisms of ^222^Rn through the porous microstructure. During the hydration phase, the formation of C-S-H gel substantially modifies the connectivity and size of the capillary pores, affecting both the diffusion coefficient of ^222^Rn and the accessibility of the recoil atoms generated by decay to the open pore network. At early curing ages, the capillary porosity is high and partially interconnected, favoring gas migration prior to decay. For this reason, as hydration reactions progress, the refinement and progressive discontinuity of the pore network reduce the effective diffusivity. This process could contribute, together with the repositioning of ^226^Ra, to the decrease in ^222^Rn emanation observed between 2 and 28 days of curing in cements containing slag and limestone. Recent studies on cement pastes with additions have shown that the ^222^Rn diffusion coefficient can vary by an order of magnitude between curing ages, depending on the porosity of the cement [46].

### 3.3. Annual Effective Doses in the Reference Dwelling

Table 5 shows the annual effective doses due to ^222^Rn inhalation and external gamma radiation in a reference dwelling for both adults and infants, for the mortars prepared with the cements studied at a curing age of 28 days. The largest contribution to the dose was due to ^222^Rn inhalation rather than external radiation. The highest effective doses corresponded to the 3 cements with GGBFS addition, as the activity concentrations and ^226^Ra content were higher in this type of cement. Therefore, the most critical value was that obtained for an infant for the CEM VI (S-L) and CEM VI (S-F) cements.

These effective dose levels are comparable to those reported in the literature for other building materials incorporating industrial by-products [31]. For instance, it has been estimated that the use of fly ash from thermal power plants as a building material can generate cumulative annual doses of between 0.1 and 0.9 mSv, reaching effective dose rates of up to 0.49 mSv/year in the case of internal doses in dwellings built with materials with a high fly ash content [47]. Therefore, the values obtained in the present study (0.17–0.32 mSv y^−1^) are safe from a radiological protection standpoint, and lie below the highest values reported for materials incorporating industrial by-products of greater radiological risk [48]. Consequently, the blast furnace slag-blended cements (S) used in this study do not pose a radiological risk compared with other building materials in which NORM materials are incorporated as supplementary cementitious materials [49].

The results shown in Table 5, obtained using the RESRAD-BUILD model, were calculated conservatively, considering that the walls, floor, and ceiling of the reference dwelling are composed entirely of the mortars considered in this study (Table 1). However, it should be noted that the mortar only occupies a wall thickness of between 2 cm and 5 cm over the structural support (concrete, brick, etc.), which does not contribute significantly to the dose. Therefore, the annual dose rates obtained should be regarded as the upper limit that could be contributed by the use of this type of mortar, although the actual value will be lower than the one obtained. In addition to material thickness, other assumptions of the RESRAD-BUILD model, such as the dwelling geometry and occupancy conditions, may influence the estimated annual effective dose rate. The design of the reference dwelling used in this study (35 m^2^, with three types of rooms) is based on the typical dimensions recommended by Directive 2013/59/Euratom. However, variations in room volume or in the distance between the receptor and the walls and floor may influence the calculation of the external dose as well as that due to ^222^Rn inhalation, since RESRAD-BUILD integrates the dose at multiple positions within each room. Similarly, the occupancy factors employed for both the adult and the infant follow the typical values of the code. Nevertheless, this type of model is largely influenced by the air exchange rate and the thickness of the materials [50]. Therefore, the annual dose rate values presented in this work should be interpreted as representative estimates for a standard reference dwelling, rather than as absolute values applicable to any real geometric or occupational configuration. Consequently, the use of this type of cement is safe from a radiological protection standpoint, making it a good option for the reuse of materials within a circular economy.

### 3.4. Summary of the Relationships Between Composition, Activity Concentration of Natural Radionuclides, and Radiological Risk

To facilitate the interpretation of the results presented in the previous sections and to provide an integrated overview of the study, Table 6 summarizes, for each cement type, the proportion of limestone (L), recycled concrete fines (F), and slag additions (S), together with the mean activity concentrations of ^226^Ra, ^232^Th, and ^40^K in the corresponding 28-day mortars, and the resulting annual effective dose for infants, classified with respect to the reference value of 50 Bq kg^−1^ for construction materials [31]. Table 6 clearly shows that although the slag-containing cements (CEM II/C-M, CEM VI (S-L), and CEM VI (S-F)) exhibit the highest natural radionuclide content among the anhydrous cements, all the corresponding 28-day cured mortars remain below the reference activity concentration of 50 Bq kg^−1^ and yield annual effective dose rates no higher than 0.32 mSv, thereby confirming their safety from a radiological standpoint and their suitability as a material for reuse within a circular economy framework.

## 4. Conclusions

The addition of limestone (L), granulated blast-furnace slag (S) and recycled concrete fines (F) does not pose a risk from a radiological protection standpoint. This conclusion is supported by the study carried out, which encompasses both the individual materials, the anhydrous cements with the addition of these three materials, and the mortars prepared with standard sand and the cements tested. These results are consistent with the european and international radiological protection legislative framework applied to building materials, confirming that the incorporation of additions regulated under EN 197-6 does not compromise the radiological safety of dwelling occupants.

The results showed that S was the individual material with the highest content of natural radionuclides, with activity concentrations of 113 ± 24 Bq kg^−1^ of ^238^U (in secular equilibrium) being obtained. However, ^210^Pb did not show secular equilibrium with the remaining radionuclides of the uranium series, owing to losses during the combustion stage in the kiln. The activity concentrations of ^40^K and the thorium-series radionuclides were below the world average values. Likewise, the lowest emanation fraction value corresponded to S, with a value of 0.0607 ± 0.0034.

The anhydrous cements with S addition (CEM II/C-M, CEM VI (S-L), and CEM VI (S-F)) showed higher concentrations of SiO_2_, Al_2_O_3_, MgO, and TiO_2_ due to the iron ore, coke, and coal burnt during their production process. Furthermore, the addition of S also resulted in these cements having uranium-series activity concentrations that were higher than the reference average value (50 Bq kg^−1^). However, it is important to note that the value of 50 Bq kg^−1^ constitutes a statistical reference level (screening level) derived from the European average for construction materials, rather than a regulatory limit or a radiological exemption threshold. Therefore, the fact that a cement exceeds this value does not necessarily imply a significant radiological risk, but should be assessed together with the estimated annual effective dose, which remains well below the dose reference level of 1 mSv y^−1^ established by national and European legislation. The principal component analysis carried out to study the relationships between the chemical composition and radiological content of the anhydrous cements again showed this behavior. On the one hand, the cements with S addition (individually and blended with L and F) showed a correlation between ^232^Th, Al_2_O_3_, and SiO_2_, whereas the cements with L and F addition showed a greater presence of K_2_O, P_2_O_5_, and ^40^K. On the other hand, the mortars in all cases had concentrations below the world average value, being lower than 25 Bq kg^−1^ for the uranium-series radionuclides and 15 Bq kg^−1^ for the thorium-series radionuclides. The ^40^K concentrations averaged 100 Bq kg^−1^, since both the clinker and the siliceous aggregate had a similar proportion of ^40^K.

The ^222^Rn emanation fractions in the anhydrous cements and mortars showed that limestone caused an increase in ^222^Rn emanation, possibly owing to the formation of carboaluminates, which could bring about changes in the distribution of ^226^Ra within the crystal structure of the mortars. On the other hand, the anhydrous cements and mortars containing recycled concrete fines addition showed greater stability in ^222^Rn emanation. However, the addition of S reflected a possible alteration in the arrangement of ^226^Ra during the hydration reactions, since the emanation fraction was lower in the anhydrous cement than in the mortars.

## Figures and Tables

**Table 1:** Composition of the cements analyzed in this study.

Cement	Clinker (%)	Limestone (L) (%)	Recycled Concrete Fines (F) (%)	Blas-Furnace Slag (S) (%)
CEM I	95–100			
CEM II/A-L	80	20		
CEM II/A-F	80		20	
CEM II/B-L	65	35		
CEM II/B-F	65		35	
CEM II/C-M	50	15	20	15
CEM VI (S-L)	35	20		45
CEM VI (S-F)	35		20	45

**Table 2:** Activity concentrations (Bq kg^−1^) of the gamma-emitting radionuclides from the natural uranium and thorium radioactive series, along with ^40^K, for the limestone (L), demolition fines (F), granulated blast-furnace slag (S), and standard sand (NA) samples. ^226^Ra was determined using both gamma-ray spectrometry (g.s.) and radiochemical separation (r.s.).

Sample	Natural Uranium Radioactive Series						Thorium Radioactive Series			^40^K
	^234^Th	^226^Ra (g.s.)	^226^Ra (r.s.)	^214^Pb	^214^Bi	^210^Pb	^228^Ac	^212^Pb	^208^Tl	
L	15.8 ± 3.7	14.1 ± 3.9	13.3 ± 2.2	15.6 ± 2.4	13.8 ± 1.1	15.7 ± 4.7	1.56 ± 0.32	<0.386	0.74 ± 0.15	23.3 ± 2.9
S	113 ± 24	104 ± 25	92 ± 11	112 ± 17	106.5 ± 6.3	<7.129	48.1 ± 3.4	50.5 ± 8.2	18.7 ± 2.3	59.2 ± 7.4
R	18.4 ± 5.3	17.6 ± 7.7	16.7 ± 2.6	14.9 ± 2.4	13.8 ± 1.3	15.8 ± 4.8	5.83 ± 0.64	5.8 ± 1.1	2.28 ± 0.49	62.8 ± 7.2
NA	6.1 ± 1.7	6.7 ± 2.1	6.8 ± 1.1	4.21 ± 0.71	4.30 ± 0.64	6.8 ± 2.3	7.20 ± 0.87	7.2 ± 1.2	2.75 ± 0.37	147 ± 13

Uncertainties are expressed for a coverage factor of k = 2.

**Table 3:** Activity concentrations of the natural radionuclides belonging to the natural uranium radioactive series (^234^Th, ^226^Ra, ^214^Pb, ^214^Bi, and ^210^Pb) and thorium series (^232^Th, ^228^Ac, ^212^Pb, and ^208^Tl), together with the activity concentration of ^40^K.

Reference	^234^Th	^226^Ra ^(1)^	^214^Pb	^214^Bi	^210^Pb	^232^Th ^(1)^	^228^Ac	^212^Pb	^208^Tl	^40^K
CEM I ^(2)^	30.3 ± 6.8	25.1 ± 3.3	28.3 ± 4.4	26.6 ± 1.9	29.4 ± 8.9	15.6 ± 3.0	15.9 ± 1.4	16.3 ± 2.7	8.7 ± 1.2	175 ± 16
CEM I (2D)	15.7 ± 6.7	13.2 ± 2.1	7.1 ± 1.2	6.54 ± 0.76	17.7 ± 6.1	7.01 ± 0.74	8.67 ± 0.92	8.8 ± 1.5	2.98 ± 0.44	124 ± 12
CEM I (28D)	12.4 ± 6.9	14.6 ± 2.3	8.7 ± 1.4	7.11 ± 0.73	18.8 ± 5.9	6.29 ± 0.75	8.51 ± 0.83	8.4 ± 1.4	3.10 ± 0.44	114 ± 11
CEM II/A-F ^(2)^	25.1 ± 8.3	25.3 ± 3.6	25.0 ± 3.9	22.7 ± 1.9	32 ± 12	12.2 ± 4.5	14.5 ± 1.4	14.6 ± 2.4	7.4 ± 1.0	144 ± 14
CEM II/A-F (2D)	13.0 ± 6.6	10.7 ± 1.7	7.1 ± 1.2	6.54 ± 0.77	18.1 ± 9.2	3.01 ± 0.82	7.77 ± 0.85	8.4 ± 1.4	2.77 ± 0.42	123 ± 12
CEM II/A-F (28D)	15.5 ± 6.3	6.8 ± 1.5	6.7 ± 1.1	5.81 ± 0.74	15.9 ± 8.6	6.67 ± 0.66	7.97 ± 0.87	8.3 ± 1.5	2.93 ± 0.39	103 ± 11
CEM II/A-L ^(2)^	25.4 ± 8.9	21.8 ± 2.9	24.6 ± 4.1	24.6 ± 2.5	27 ± 12	17.5 ± 4.1	12.9 ± 2.1	14.5 ± 2.6	7.6 ± 1.4	135 ± 15
CEM II/A-L (2D)	13.0 ± 3.1	11.3 ± 1.9	8.3 ± 1.3	8.06 ± 0.77	23.4 ± 8.4	5.96 ± 0.64	7.98 ± 0.80	8.1 ± 1.3	2.98 ± 0.42	120 ± 11
CEM II/A-L (28D)	16.7 ± 7.1	9.8 ± 1.7	6.6 ± 1.1	6.39 ± 0.76	13.0 ± 8.6	5.29 ± 0.62	7.63 ± 0.89	7.9 ± 1.3	2.76 ± 0.42	111 ± 11
CEM II/B-F ^(2)^	43 ± 14	22.9 ± 3.1	22.7 ± 3.9	22.7 ± 3.0	32 ± 13	10.8 ± 1.4	12.8 ± 1.7	13.2 ± 1.2	7.4 ± 1.9	129 ± 40
CEM II/B-F (2D)	13.3 ± 3.4	8.6 ± 1.5	7.1 ± 1.5	6.8 ± 1.0	20.4 ± 9.1	7.11 ± 0.66	6.91 ± 0.65	8.2 ± 1.5	2.95 ± 0.60	116 ± 12
CEM IIB-F (28D)	11.8 ± 2.9	8.5 ± 1.6	8.2 ± 1.3	7.55 ± 0.74	12.3 ± 4.0	5.37 ± 0.62	7.33 ± 0.75	8.0 ± 1.3	3.01 ± 0.42	108 ± 10
CEM II/B-L ^(2)^	25.3 ± 5.5	22.5 ± 3.3	23.9 ± 3.7	22.1 ± 1.5	28.2 ± 8.3	8.1 ± 2.4	10.85 ± 0.92	11.0 ± 1.8	5.89 ± 0.78	112 ± 10
CEM II/B-L (2D)	10.2 ± 5.7	9.2 ± 1.5	6.8 ± 1.2	6.92 ± 0.78	15.3 ± 7.1	6.43 ± 0.62	7.82 ± 0.85	8.5 ± 1.4	2.82 ± 0.42	115 ± 11
CEM II/B-L (28D)	11.7 ± 7.6	10.0 ± 1.7	8.0 ± 1.6	6.8 ± 1.8	17.4 ± 9.3	6.4 ± 1.2	6.38 ± 0.65	7.9 ± 1.6	2.85 ± 0.64	107 ± 12
CEM II/C-M ^(2)^	39.7 ± 8.7	33.0 ± 4.0	35.4 ± 5.4	33.1 ± 2.2	27 ± 10	16.1 ± 1.7	17.4 ± 1.4	16.9 ± 2.8	9.6 ± 1.2	106 ± 10
CEM II/C-M (2D)	15.8 ± 3.6	14.8 ± 2.3	10.6 ± 1.7	9.83 ± 0.87	14.2 ± 4.5	11.2 ± 1.8	9.10 ± 0.85	8.8 ± 1.4	3.24 ± 0.45	117 ± 11
CEM II/C-M (28D)	13.9 ± 3.8	17.7 ± 2.4	8.6 ± 1.4	7.96 ± 0.84	11.9 ± 4.4	9.59 ± 0.82	9.01 ± 0.91	9.4 ± 1.6	3.49 ± 0.49	111 ± 11
CEM VI (S-F) ^(2)^	60 ± 13	55.2 ± 6.3	58.9 ± 9.0	54.5 ± 3.4	14.4 ± 5.0	30.2 ± 3.9	27.4 ± 2.0	27.6 ± 4.5	9.7 ± 1.2	120 ± 11
CEM VI (S-F) (2D)	22.0 ± 8.2	15.9 ± 2.3	12.5 ± 2.0	11.1 ± 1.0	13.5 ± 8.4	10.3 ± 1.0	11.5 ± 1.1	10.9 ± 1.8	4.14 ± 0.58	113 ± 11
CEM VI (S-F) (28D)	24.9 ± 6.0	19.8 ± 2.9	12.9 ± 2.1	12.4 ± 1.1	10.2 ± 4.2	9.7 ± 1.0	10.4 ± 1.1	11.9 ± 2.0	4.10 ± 0.58	119 ± 12
CEM VI (S-L) ^(2)^	62 ± 17	53.5 ± 6.2	61 ± 10	56.1 ± 4.8	18.3 ± 6.3	24.5 ± 2.0	28.5 ± 2.7	29.0 ± 4.9	15.4 ± 2.5	96 ± 16
CEM VI (S-L) (2D)	22.3 ± 5.0	16.7 ± 2.6	15.4 ± 2.4	13.9 ± 1.2	10.6 ± 3.7	9.04 ± 0.91	10.9 ± 1.0	11.3 ± 1.9	4.15 ± 0.57	115 ± 11
CEM VI (S-L) (28D)	23.6 ± 5.7	14.1 ± 2.3	13.4 ± 2.1	11.8 ± 1.1	11.6 ± 4.6	8.81 ± 0.82	11.5 ± 1.1	11.5 ± 1.9	4.20 ± 0.59	109 ± 11

Uncertainties are expressed for a coverage factor of k = 2. ^(1) 226^Ra and ^232^Th were determined by radiochemical separation. ^(2)^ These are the anhydrous cements described in Table 1.

**Table 4:** Emanation fractions for the individual anhydrous cements and for the mortars prepared at curing ages of 2 and 28 days.

Cement	Anhydrous	2 Days	28 Days
CEM I	0.519 ± 0.046	0.400 ± 0.039	0.471 ± 0.042
CEM II/A-L	0.756 ± 0.088	0.720 ± 0.072	0.923 ± 0.079
CEM II/A-F	0.480 ± 0.046	0.479 ± 0.047	0.476 ± 0.032
CEM II/B-L	0.358 ± 0.041	0.610 ± 0.045	0.720 ± 0.054
CEM II/B-F	0.476 ± 0.055	0.406 ± 0.036	0.487 ± 0.046
CEM II/C-M	0.272 ± 0.029	0.446 ± 0.039	0.277 ± 0.023
CEM VI (S-L)	0.191 ± 0.021	0.562 ± 0.036	0.420 ± 0.037
CEM VI (S-F)	0.310 ± 0.035	0.586 ± 0.037	0.610 ± 0.049

Uncertainties are expressed for a coverage factor of k = 2.

**Table 5:** Annual effective doses (mSv) due to ^222^Rn inhalation and external gamma radiation in a reference dwelling (infants and adults) for the mortars prepared with the cements studied at a curing age of 28 days.

Mortar Type	^222^Rn Infant (mSv)	^222^Rn Adult (mSv)	Gamma Infant (mSv)	Gamma Adult (mSv)	Effective Dose Infant (mSv)	Effective Dose Adult (mSv)
CEM I	0.14	0.14	0.11	0.09	0.25	0.23
CEM II/A-L	0.10	0.10	0.12	0.10	0.22	0.20
CEM II/A-F	0.10	0.12	0.07	0.07	0.17	0.19
CEM II/B-L	0.11	0.11	0.09	0.09	0.20	0.20
CEM II/B-F	0.12	0.12	0.08	0.07	0.20	0.19
CEM II/C-M	0.18	0.18	0.10	0.08	0.28	0.26
CEM VI (S-L)	0.19	0.18	0.13	0.12	0.32	0.30
CEM VI (S-F)	0.15	0.14	0.15	0.12	0.30	0.26

**Table 6:** Summary of the percentage composition of limestone, recycled concrete fines and blast-furnace slag, activity concentration of ^226^Ra, ^232^Th and ^40^K along with the effective dose for infants for the mortars manufactured with the cements studied in this work for a curing time of 28 days.

Cement	L (%)	F (%)	S (%)	^226^Ra (Bq kg^−1^)	^232^Th (Bq kg^−1^)	^40^K (Bq kg^−1^)	Effective Dose Infant (mSv)
CEM I	–	–	–	14.6	6.29	114	0.25
CEM II/A-L	20	–	–	9.8	5.29	111	0.22
CEM II/A-F	–	20	–	6.8	6.67	103	0.17
CEM II/B-L	35	–	–	10.0	6.40	107	0.20
CEM II/B-F	–	35	–	8.5	5.37	108	0.20
CEM II/C-M	15	20	15	17.7	9.59	111	0.28
CEM VI (S-L)	20	–	45	14.1	8.81	109	0.32
CEM VI (S-F)	–	20	45	19.8	9.70	119	0.30

## Data Availability

The original contributions presented in this study are included in the article. Further inquiries can be directed to the corresponding author.
